# Supplementary material for: Factors associated with viral load re-suppression after enhanced adherence counseling among people living with HIV with an initial high viral load result in selected Nigerian states
Source: PLOS Glob Public Health. 2024 Nov 22;4(11):e0002876. doi: 10.1371/journal.pgph.0002876 (PMC11584108; doi:10.1371/journal.pgph.0002876)
Supplement: Attachment — Submitted filename: Response to Reviewers.docx [file pgph.0002876.s002.docx]

**Response to Reviewers**

Dear Reviewers,

Thank you for your feedback on our manuscript entitled Factors associated with viral Load re-suppression after enhanced adherence counseling among people living with HIV with an Initial high viral load result in RISE-supported facilities in five States in Nigeria. We appreciate the time and effort you have dedicated to evaluating our work. Below, we address each of your comments and suggestions:

Reviewer #1:

1. Title: We have removed the “RISE” acronym in the title.

2. Abstract: We have restructured the abstract to improve clarity and separate background information from methods. We have also replaced the term "Recipients of HIV Care (ROC)." to People living with HIV (PLHIV)

3. Results: We have provided absolute numbers before proportions and rephrased the sentence regarding adolescents and PLHIV on second-line regimens for clarity.

4. Study design: We have specified the type of descriptive study conducted.

5. Program data and setting: We have also revised these sections to enhance clarity and understanding.

6. Study population: Similarly, we will ensure that this section provides distinct information from the study setting.

7. Data analysis: We have clarified the description of the data analysis methods used.

8. Results: We have also ensured that the results section is presented more clearly and aligned with the study objectives.

9. General comments: We have removed non-standard acronyms like "ROC," and ensure compliance with STROBE guidelines.

Reviewer #2:

1. Methods: We have provided context on how usual HIV care is provided in Nigeria and describe the RISE-supported Health Facilities and the EAC intervention in more detail.

2. Results: We have also included information on baseline Viral load suppression and addressed questions regarding patients who received no EAC and those who completed fewer sessions.

3. Discussion: We discussed potential reasons for differences in study results compared to other studies and exercised caution in claiming effectiveness without baseline or controls.

Thank you once again for your valuable feedback.

Sincerely,

Obasa Gbenga Benjamin
